# Gut microbiota and eye diseases: A review

**DOI:** 10.1097/MD.0000000000039866

**Published:** 2024-09-27

**Authors:** Yue Zhao, Peijin Qiu, Ting Shen

**Affiliations:** a School of Public Health, Hangzhou Medical College, Hangzhou, China; b Eye Center, The Second Affiliated Hospital, Zhejiang University School of Medicine, Hangzhou, Zhejiang, China.

**Keywords:** eye disease, gut microbiota, ocular surface, treatment

## Abstract

Recent studies reveal that alterations in gut microbiota play a significant role in the progression of various diseases, including those affecting the eyes. The association between gut microbiota and eye health is an emerging focus of research. This review seeks to summarize the connection between the gut microbiome and specific eye conditions, such as ocular surface diseases, funduscopic disorders and immune-mediated eye diseases. Gut microbiota may influence these conditions by regulating the immune system or altering metabolites, thereby contributing to disease development. Strategies like probiotics, antibiotics, dietary modifications, and fecal transplants show promise in addressing these issues. This review examines how the gut microbiome may be linked to the pathogenesis of eye diseases, providing fresh therapeutic perspectives for ophthalmology.

## 1. Introduction

The human body harbors numerous bacteria, all of which significantly impact our overall health.^[1]^ Among these, gut microbes are particularly vital.^[2]^ These microbes are heterogeneous and vary across individuals. The intestinal microbiota is a dynamic system that begins forming early in life and is easily influenced by external factors such as environment, diet, and lifestyle, eventually stabilizing in adulthood.^[3]^ It plays a crucial role in maintaining the integrity of the intestinal barrier, influencing host nutrient metabolism, participating in immune regulation, and combating pathogens.^[4,5]^ The intestinal microbiota consists of bacteria, fungi, archaea, viruses, and other microorganisms, with bacteria being the most predominant.^[6]^ At the phylum level, Bacteroidetes, Actinomycetes, Firmicutes, and Proteobacteria are the most prevalent.^[7]^ When the gut microbiota is dysbiotic, its diversity and abundance diminish, leading to impaired physiological functions and affecting extraintestinal organs such as the nervous and immune systems.^[8,9]^ The gut microbiota is closely linked to systemic diseases, influencing their onset and progression. Increasing evidence suggests that gut microbiome dysregulation is associated with ocular diseases, including diabetic retinal disease, age-related macular degeneration, glaucoma, and autoimmune uveitis.^[10–12]^ The primary aim of this review is to elucidate the relationship between gut microbiota and ocular diseases and to propose potential therapeutic strategies, offering new insights for the prevention and treatment of eye diseases.

## 2. Ocular surface diseases

### 2.1. Dry eye

Dry eye is a chronic ocular surface disorder caused by tear film instability or an imbalance in the ocular surface microenvironment. It is primarily categorized into aqueous deficiency and evaporative dry eye. Sjögren’s syndrome (SS) dry eye, a subtype of aqueous deficiency, is a chronic autoimmune condition affecting the salivary and lacrimal glands, often presenting with dry mouth and eyes. Primary Sjögren’s syndrome (pSS) is more prevalent.^[13]^ While the etiology of pSS remains unclear, genetic and environmental factors likely contribute to its pathogenesis.^[14]^

The gut microbiome undergoes significant changes in dry eye patients. Cano-Ortiz et al^[15]^ found that, compared to healthy controls, pSS patients had reduced intestinal flora richness and diversity, with increased Bacteroidetes and Proteobacteria, and decreased Firmicutes and Actinobacteria. These findings are consistent with recent studies.^[16]^ Van der Meulen et al^[17]^ also observed reduced richness in pSS patients, though they found no significant difference in diversity. This may be due to population heterogeneity. While some studies noted decreased Praxellosis and increased Prevotella in SS patients, others reported a reduction in Prevotella.^[18,19]^ A lower Firmicutes/Bacteroidetes ratio, a key marker of gut microbiota imbalance, has been consistently observed in SS patients.^[20,21]^ Reduced abundance and diversity in gut flora are linked to increased inflammatory biomarkers and a compromised intestinal barrier, promoting pathogenic bacteria and inflammation.^[19]^ These factors are critical for gut and overall health.

The relationship between gut microbiota and dry eye may be mediated through the modulation of T cell subsets and the subsequent inflammatory response. Zaheer et al^[22]^ observed that CD4 + T cells isolated from adoptively transferred sterile female CD25 knockout (CD25KO) mice induced more severe SS than those from conventional CD25KO mice. In their study, germ-free CD25KO mice exhibited reduced goblet cell density and severe corneal barrier damage, indicating that commensal bacteria play a crucial role in inducing CD4 + T cell differentiation. These bacteria regulate inflammation by activating different T cell subsets, thereby influencing the onset and progression of inflammation. CD25KO mice spontaneously develop Sjögren’s-like inflammation, consistent with C57BL/6j mice. Interestingly, the pathogenicity of CD4 + T cells was reduced when fecal bacteria from normal C57BL/6j mice were transplanted into sterile CD25KO mice, suggesting a modulatory effect of gut microbiota on inflammation.^[23]^ Antibiotic treatment in naive mice significantly altered gut microbiota composition, with an increase in Proteobacteria and Firmicutes and a decrease in Bacteroidetes.^[24]^ These changes in gut microbiota may disrupt immune regulation, thereby influencing the development and progression of inflammation.

Recently, Moon et al proposed the “gut dysbiosis–ocular surface–lacrimal gland axis” hypothesis, which suggests that alterations in intestinal microbiota contribute to the onset and progression of dry eye disease (DED). These changes may trigger inflammation, affecting the lacrimal glands and corneas through neural, endocrine, or immune pathways, leading to ocular inflammation, reduced tear secretion, and diminished tear quality, ultimately causing DED.^[25]^ This hypothesis offers a profound understanding of the gut microbiota’s role in DED, indicating that improving intestinal flora could be a potential treatment strategy. Studies have shown that IRT5 probiotics can alleviate dry eye symptoms, aligning with previous findings.^[26,27]^ Recent research reveals that Lactobacillus plantarum NK151 and Bifidobacterium bifidum NK175 reduce dry eye symptoms by reshaping the gut microbiota and balancing anti-inflammatory and pro-inflammatory cytokines.^[28]^ Kawashima et al^[29]^ found that dietary supplements containing Enterococcus faecium WB2000 combined with lactoferrin improved dry eye symptoms, while Connell et al^[30]^ demonstrated that oral lactoferrin maintained tear secretion in a dry eye mouse model. Additionally, emerging studies suggest that nanomedicine offers promising treatments for dry eyes. Numerous clinical trials are underway for nanotherapeutics, including nanoemulsions, nanosuspensions, liposomes, and micelles, some of which have received Food and Drug Administration approval as novel therapies for DED.^[31]^

### 2.2. Infectious keratitis

Infectious keratitis is a corneal tissue infection caused by various pathogens, including fungi, bacteria, viruses, and parasites. When eye infections are left untreated, blindness may occur.^[32]^Previous studies have linked gut microbiota to both fungal and bacterial keratitis.

Kalyana Chakravarthy et al^[33]^ were the first to identify intestinal dysbiosis in fungal keratitis (FK) patients compared to healthy controls (HC) within an Indian cohort. They found that FK patients had a higher presence of pathogenic bacteria, such as Treponema and Bacteroides fragilis, while healthy individuals had more anti-inflammatory bacteria like Clostridium, Streptococcus, and Spirillum. This imbalance suggests that FK may be driven by an increase in harmful bacteria and a decrease in beneficial, anti-inflammatory bacteria. Although bacteria are more abundant in the gut, fungi also play a significant role in human health.^[34]^ Jayasudha et al later observed that both bacterial and fungal diversity and abundance were reduced in bacterial keratitis (BK) patients compared to HC, which aligns with earlier findings in patients with inflammatory bowel disease who also showed reduced fecal fungal flora.^[35,36]^ Moreover, Kalyana Chakravarthy et al^[33]^ found significant differences in the bacterial microbiomes among HC, BK patients taking antibiotics, and BK patients not on antibiotics, indicating that while antibiotics contribute to these differences, they are not the only factor. A healthy gut microbiome is essential for overall health, and some studies suggest it may provide immune protection against keratitis caused by Pseudomonas aeruginosa in Swiss Webster mice.^[37]^ However, the link between gut microbiota and keratitis remains under-researched and requires more scientific evidence.

## 3. Retinal and macular diseases

### 3.1. Age-related macular degeneration

Age-related macular degeneration (AMD) is a chronic retinal disease that primarily affects the macular region and is the leading cause of irreversible vision loss among the elderly in developed countries.^[38]^ As the global population ages, the incidence of AMD is projected to rise, reaching 288 million cases by 2040, posing a significant public health challenge.^[39]^ AMD is a multifactorial disease, with smoking, lifestyle, age, obesity, nutrition, and genetics all contributing to its development, with age being the most prominent risk factor.^[40]^

Zysset-Burri et al^[41]^ observed that Bacteroides were abundant in healthy individuals, while Firmicutes were more prevalent in AMD patients. This finding indicates a close association between gut microbial changes and the development of AMD. Lin^[42]^ further discovered that dysbiosis in the gut microbiota led to an increase in the abundance of Prevotella, Holdemanella, and Desulphurvibrio in AMD patients, while Tremillum, Blautia, and Dorea were less abundant. Additionally, it was found that Anaerobaculum, Ruminococcus, and Eubacterium were relatively enriched in AMD patients, whereas Bacteroides were more prevalent in the control group.^[43]^ A recent study using 2-sample Mendelian randomization (MR) analysis identified Anaerotruncus, Candidatus Soleaferrea, and an unknown genus id.2071 as protective factors against AMD, while the Eubacterium Oxidoreducens group, Faecalibacterium, and Ruminococcaceae UCG-011 were identified as risk factors.^[44]^

Rowan et al^[45]^ introduced the concept of the gut-retinal axis, highlighting the interaction between the gut and retina. Changes in gut microbiota can influence the visual system’s health and disease states through mechanisms such as immune regulation, neural signaling, and metabolic control. However, due to the variability of gut microbiota, the understanding of the gut-retinal axis is still in its infancy. Researchers have applied MR analysis to reduce confounding factors and enhance the validity of studies on the gut-retinal connection.^[46]^ MR is increasingly used to explore the causal links between gut flora and eye diseases.^[47]^ Liu et al^[40]^ were the first to identify, through MR analysis, the involvement of Rhodospirillaceae in ocular diseases, confirming its role as a risk factor for AMD within the gut-retinal axis. This study offers new insights into leveraging gut microbiota for AMD prevention.

The current treatment options for AMD are limited. Management of dry AMD primarily focuses on preventing or controlling risk factors, while wet AMD is treated with anti-VEGF drugs. Recent findings suggest that intravitreal administration of avacincaptad pegol, a C5 inhibitor, can reduce geographic atrophy in AMD patients.^[48]^ Dietary choices also influence the gut microbiome differently. Andriessen et al^[49]^ discovered that a high-fat diet alters gut microbiota and exacerbates choroidal neovascularization. Conversely, vitamins C and E, along with higher intake of omega-3 fatty acids, have been shown to lower the risk of AMD.^[50]^ Currently, the only proven method to slow AMD progression is the use of oral Age-Related Eye Disease Study 2 supplements.^[51]^ Parker et al^[52]^ found that aging increases intestinal permeability, but transplanting microbiota from young donors can reduce this permeability, thereby lowering inflammation levels.

### 3.2. Diabetic retinal disease

Diabetes mellitus (DM) is a chronic disease characterized by persistently elevated blood glucose levels. The global incidence of DM is steadily rising, with the International Diabetes Federation projecting an increase in the number of DM patients from 451 million in 2017 to 693 million by 2045.^[53]^ Diabetic retinopathy (DR), a microvascular complication of DM, is the leading cause of blindness among working-age individuals worldwide.^[54]^ However, not all DM patients develop DR; the incidence is approximately 95% in type 1 DM and 60% in type 2 DM.^[55]^ The precise pathogenesis of DR remains unclear, but it is influenced by various factors, including the duration and progression of DM, poor glycemic control, oxidative stress, inflammation, and angiogenesis.^[56]^

Recent studies have increasingly linked gut microbiota disturbances to DR. Prasad et al^[57]^ demonstrated that an imbalance in gut flora induces retinal inflammation and impairs barrier function in Akita mice, highlighting the connection between DR and microvascular damage. Comparative analyses have shown that DR patients exhibit reduced levels of beneficial bacteria, such as Actinobacteria and Bacteroidetes, with corresponding decreases in Bifidobacteria and Lactobacillus.^[8,58]^ Interestingly, while bifidobacteria are typically seen as beneficial, they were found to be elevated in DR and DM patients, alongside a reduction in Escherichia-Shigella, Faecalibacterium, and Clostridium.^[10]^ Significant differences in gut microbiota composition have been noted between DR, healthy controls, and DM patients, with DR patients showing an increase in Faecalibacterium and Prevotella, and a decrease in Akkermansia.^[59]^ Additionally, MR studies have identified Christensenellaceae and Peptococcaceae as protective factors against DR, while Eubacterium rectale, Adlercreutzia, and Ruminococcaceae UCG-011 are linked to increased DR risk.^[60]^ Further research is needed to explore these relationships at the species level.

The “gut-retinal axis” plays a crucial role in the development of DR. Caspi et al^[61]^ broke the previous belief that live microorganisms exist within the eye, revealing that the inner eye remains sterile while the outer eye is exposed to environmental microbes. Gut dysbiosis increases intestinal permeability, allowing pro-inflammatory substances and bacteria to enter the circulatory system, thereby triggering systemic inflammation. This inflammatory state can have severe repercussions on the retina. Current mechanisms underlying the “gut-retina” hypothesis include impaired intestinal barrier function, activation of Stimulator of Interferon Genes, deficiency in angiotensin-converting enzyme 2, the influence of intestinal microbial metabolites, and the production of lipopolysaccharides.^[62–66]^

However, the treatment options for DR remain limited, making the modulation of gut microbiota a potential new therapeutic direction. Huang et al^[67]^ concluded that butyrate supplementation improved retinal visual function in diabetic mice, suggesting its potential to slow DR progression. Other studies have demonstrated that oral administration of Ang-(1-7)-L or angiotensin-converting enzyme 2 to diabetic mice can alleviate DR development.^[68]^ Dietary habits also influence gut microbiota, with certain diets potentially offering beneficial effects.^[69]^ Beli et al^[70]^ discovered that intermittent fasting improved the integrity of the intestinal barrier and altered the gut microbiota composition in db/db mice, leading to an enrichment of Firmicutes and a reduction in Bacteroides. Additionally, studies have shown that long-term use of metformin can reduce the incidence of DR.^[71]^ Recent research observed abnormal vascular morphology in the deep capillary layer around the optic disc in children with T1DM, highlighting the importance of monitoring peripapillary deep capillary in DR prevention and treatment.^[72]^

### 3.3. Retinal artery occlusion

Retinal artery occlusion (RAO) is an ischemic condition of the inner retina caused by obstruction of blood flow in the retinal artery. The pathogenesis of RAO is complex, primarily involving atherosclerosis and thrombosis, with embolism often originating from plaque formation in major arteries.^[73]^ Studies have demonstrated a close link between gut microbiota and atherosclerosis, with gut microbes contributing to the disease’s progression through the regulation of host metabolism and inflammation.^[74]^ Thus, it can be inferred that alterations in gut microbiota may also be associated with the onset of RAO.

Zysset-Burri et al^[75]^ identified a link between the intestinal microbiome and non-arterial RAO. In RAO patients, compared to the control group, there was an enrichment of Bifidobacterium, Actinomyces, and fecal Bacteroides, along with a significant increase in the concentration of trimethylamine N-oxide (TMAO), a gut microbial metabolite. Recent research has established a strong connection between TMAO and hypercholesterolemia, a condition that contributes to the development of atherosclerosis.^[76]^ Notably, 2 studies have confirmed the presence of bacterial DNA, primarily from Proteobacteria and Actinobacteria, in human atherosclerotic plaques.^[77,78]^ Shin et al^[79]^ found that an increased prevalence of Proteobacteria serves as an early indicator of gut microbiota imbalance, allowing for earlier detection of microbial alterations. As atherosclerosis progresses, it can lead to the narrowing of the vascular lumen, particularly affecting the retinal artery, resulting in ischemia and occlusion. Atherosclerosis and cardiovascular disease (CVD) are mutually reinforcing conditions. Studies have shown that inhibiting TMAO can treat CVD, likely by preventing atherosclerosis. Since RAO shares the same etiology and pathology as CVD, inhibiting TMAO could also be a therapeutic approach for RAO.^[80]^

## 4. Immune-mediated eye diseases

### 4.1. Behçet’s disease

Behcet’s disease (BD) is a chronic and rare immune-mediated multi-system inflammatory condition, commonly affecting the eyes in the Mediterranean and Middle East regions.^[81]^ BD is a significant cause of blindness due to ocular complications. The precise pathogenesis of BD remains unclear, though it is believed to be influenced by environmental, genetic, and infectious factors.^[82]^ The disease typically manifests between the ages of 20 and 40, with an equal distribution between men and women. However, male gender, earlier onset, and involvement of multiple organs at diagnosis are associated with a more severe progression of the disease.^[83]^

Shimizu et al^[84]^ discovered that, in comparison to HC, BD patients exhibited an enrichment of Lactobacillus and Bifidobacteria, along with a decrease in Prevotella, while Butyricobacteria were more prevalent in HC. This aligns with previous findings suggesting that Prevotella is a primary dysregulated target in immune diseases.^[85]^ The diversity of intestinal flora in BD patients is notably reduced. Three studies consistently found a significant increase in Bifidobacterium and Eggerthella, and a marked decrease in Macromonas, Butyricvibrio, Prevotella, and Lachrillum in BD patients compared to HC.^[86–88]^ Ye et al^[89]^ reported an increase in sulfate-reducing bacteria (SRB) and a decrease in butyrate-producing bacteria (BPB) in BD patients, with BD showing a positive correlation with SRB and a negative correlation with BPB. SRB, known as pro-inflammatory bacteria, are implicated in the pathogenesis of various immune diseases. Conversely, BPB play a crucial role in maintaining gut health by producing short-chain fatty acids, particularly butyrate and propionate. A reduction in butyrate leads to abnormal T cell differentiation, triggering pathological T effector responses and subsequent inflammation.^[90]^ Wang et al^[91]^ were the first to establish a connection between disturbances in intestinal microflora and neutrophil activation in BD patients. When the immune system is compromised, inflammatory factors are released, triggering the activation of neutrophils and helper T17 (Th17) cells. Intriguingly, Shimizu et al^[92]^ also observed an increase in Th17 cells in BD patients.

### 4.2. Uveitis

Uveitis (UVT) is an inflammatory eye disease that can impact various parts of the eye, including the iris, retina, vitreous, choroid, and ciliary body, leading to visual impairment. UVT is categorized into 2 types based on its causative factors: infectious and noninfectious.^[93]^ The latter, primarily autoimmune in nature, is recognized as a significant contributor to vision impairment and ocular complications in developed nations.^[94]^ While different types of UVT have diverse etiologies, factors such as infections, environmental influences, and autoimmune reactions all play a role in the disease’s onset.^[95]^

UVT is an ocular inflammatory disease that has garnered significant attention regarding its pathogenesis. Lu et al^[96]^ identified the gut microbiome as a crucial factor in the pathogenesis of UVT by examining global incidence trends over the past decade. Research has revealed substantial differences in gut microbiome composition between UVT patients and healthy individuals. Wang et al^[97]^ compared the gut microbiota of Behcet’s uveitis and Vogt–Koyanagi–Harada disease patients with that of healthy controls, finding a reduction in Eubacterium hallii, Dorea longicatena, and Ruminococcus obeum in Vogt–Koyanagi–Harada and Behcet’s uveitis patients. Jayasudha et al were the first to demonstrate that the gut microbiota in UVT patients from an Indian cohort differed significantly from that of healthy controls, with a notable decrease in anti-inflammatory bacteria and an increase in pro-inflammatory and pathogenic bacteria, such as Faecalis, Bacteroides and Trichomonas. Prevotella and Streptococcus were found to be abundant.^[98]^ In a subsequent study, Jayasudha et al^[99]^ identified, for the first time, a significant dysbiosis in the intestinal fungal communities of UVT patients, with reduced richness and diversity compared to healthy individuals. These studies enhance our understanding of the relationship between gut microbiota and UVT, suggesting that the gut microbiome could be explored as a potential therapeutic target.

Gut microbiota may play a significant role in the pathogenesis of UVT through four interconnected mechanisms: First, retinal-specific T cells can be activated by antigens from the gut microbiome, as retinal antigens are sequestered within the eye’s immune-privileged environment.^[100]^ Second, disruption of the intestinal barrier increases permeability, allowing microbial products to translocate and potentially cause eye inflammation through molecular mimicry.^[101]^ Third, an imbalance in intestinal immune homeostasis, particularly between Th17 and Treg cells, is crucial in UVT development, as a healthy gut maintains a dynamic balance between these cells.^[102]^ Finally, a reduction in beneficial microbial metabolites like short-chain fatty acids can lead to inflammatory diseases by disrupting immune regulation and intestinal mucosal protection.^[103]^ These 4 mechanisms, working in concert, contribute to the onset and progression of UVT.

Treatment for uveitis primarily involves immunosuppressants like methotrexate and mycophenolate mofetil, which have been shown to positively affect the gut microbiome in experimental autoimmune uveitis.^[104]^ Fecal microbiota transplantation offers potential for restoring gut homeostasis, though its efficacy in UVT remains unproven and is still under investigation.^[105]^ Antibiotics, such as minocycline, have demonstrated benefits in animal models, though their effect on UVT is not solely due to anti-inflammatory properties.^[106]^ Combination antibiotic therapy may enhance treatment outcomes, but long-term benefits are uncertain.^[107]^ Probiotics also show promise, particularly in promoting Treg cell differentiation to regulate immune balance, with specific strains like IRT-5 and Lactobacillus reuteri showing potential in mitigating UVT progression.^[108]^

## 5. Summary and outlook

Emerging evidence increasingly suggests a significant association between gut microbiota and various eye diseases. In ocular surface disorders, gut microbiota influences the occurrence of these conditions by modulating tear secretion. In retinal diseases, alterations in the gut microbiome compromise the retinal barrier, contributing to ocular pathology. Immune-mediated eye diseases are closely linked to imbalances in intestinal flora, primarily through abnormal immune system activation. Thus, the gut microbiota plays a pivotal role in the onset and development of ocular diseases. The prospect of manipulating gut microbiota opens new avenues for innovative approaches in the treatment and prevention of eye diseases, potentially leading to more effective and targeted therapies in the future.

## Acknowledgments

Supported by the Research and Development Plan of Zhejiang Science and Technology Department (No. 2023C03089).

## Author contributions

**Funding acquisition:** Peijin Qiu.

**Writing – original draft:** Yue Zhao.

**Writing – review & editing:** Peijin Qiu, Ting Shen.
